# Memory-Enhancing Effects of *Origanum majorana* Essential Oil in an Alzheimer’s Amyloid beta1-42 Rat Model: A Molecular and Behavioral Study

**DOI:** 10.3390/antiox9100919

**Published:** 2020-09-26

**Authors:** Paula Alexandra Postu, Dragos Lucian Gorgan, Oana Cioanca, Manuela Russ, Stefan Mikkat, Michael Otto Glocker, Lucian Hritcu

**Affiliations:** 1 Department of Biology, Faculty of Biology, Alexandru Ioan Cuza University of Iasi, 700506 Iasi, Romania; paula.postu@iroiasi.ro (P.A.P.); lucian.gorgan@uaic.ro (D.L.G.); 2 Center for Fundamental Research and Experimental Development in Translation Medicine—TRANSCEND, Regional Institute of Oncology, 700483 Iasi, Romania; 3 Department of Pharmacognosy, Faculty of Pharmacy, “Grigore T. Popa” University of Medicine and Pharmacy, 700115 Iasi, Romania; oana.cioanca@gmail.com; 4 Proteome Center Rostock, Medical Faculty and Natural Science Faculty, University of Rostock, 18059 Rostock, Germany; manuela.russ@med.uni-rostock.de (M.R.); stefan.mikkat@med.uni-rostock.de (S.M.); 5 Core Facility Proteome Analysis, Rostock University Medical Center, D-18057 Rostock, Germany

**Keywords:** *Origanum majorana* L., essential oil, amyloid beta peptide, memory, oxidative stress, proteome analysis, Alzheimer’s disease

## Abstract

*Origanum* L. (Lamiaceae) is an important genus of medicinal and aromatic plants used in traditional medicine since ancient times as culinary herbs and remedies. The aim of the present study was to evaluate the chemical composition, as well as the biochemical and cellular activities of freshly prepared *Origanum majorana* L. essential oil (OmEO) in an Alzheimer’s disease (AD) amyloid beta1-42 (Aβ1-42) rat model. OmEO (1% and 3%) was inhaled for 21 consecutive days, while Aβ1-42 was administered intracerebroventricularly to induce AD-like symptoms. Our data demonstrate that OmEO increased antioxidant activity and enhanced brain-derived neurotrophic factor (BDNF) expression, which in concert contributed to the improvement of cognitive function of animals. Moreover, OmEO presented beneficial effects on memory performance in Y-maze and radial arm-maze tests in the Aβ1-42 rat AD model.

## 1. Introduction

Aromatherapy is defined as the controlled use of concentrated essential oils extracted from herbs, flowers, and other plant parts for therapeutic or preventive purposes [1]. The main routes of essential oils delivery are orally, dermally (aromatherapy massage) and olfactory administration (direct and indirect inhalation aromatherapy) [2]. Essential oils are mainly known for pharmacological properties such as antifungal, antiviral, antimicrobial [3], and antioxidant activity [4]. In recent years, several authors have highlighted aromatherapy to be supportive in relieving pain [5,6,7], reducing stress, and reinstalling emotional well-being [8] and also with alleviating anxiety [9] and depression [10].

*Origanum majorana* L. (Lamiaceae), commonly known as marjoram, is a herb native to the Mediterranean region and cultivated in many countries of Asia, North Africa, and Europe [11]. Marjoram presents a strong and pleasant fragrance and is widely used as a spice. In folk medicine, this herb has been used as a remedy against various ailments such as asthma, indigestion, headache, and rheumatism [12]. Different authors have reported marjoram’s antibacterial and antiviral properties [13,14], antioxidant [15] and anti-acetylcholinesterase (AChE) activities [16], and also anti-metastatic and anti-tumor growth effects [17]. Recently, a group of researchers demonstrated that *Origanum majorana* essential oil (OmEO) exerted antidepressant-like effects by modulating noradrenergic, dopaminergic, and serotonergic systems [18]. Another group of researchers showed that OmEO alleviated anxiety in bruxistic patients and claimed that this essential oil might represent a safe alternative for benzodiazepine treatment [19]. Moreover, *O. majorana* possesses anti-inflammatory properties [20]. An in vitro study performed by Arranz et al. [21] revealed that OmEO exerts anti-inflammatory activity through the inhibition of pro-inflammatory cytokine secretion, such as tumor necrosis factor-alpha (TNF-α), interleukin 1β (IL-1β), and interleukin 6 (IL-6) in activated macrophages.

Amyloidosis is a condition characterized by the deposition of insoluble proteins in the extracellular space of various tissues. The most frequent types of amyloidosis are related to amyloid β (Aβ) deposition, which is characteristic of neurological conditions such as aging, traumatic brain injuries, or AD [22]. Aβ peptide is an internal processing product generated through serial proteolytic cleavages of the amyloid precursor protein (APP) [23,24]. In humans, the soluble form of this peptide is produced and released in low amounts during normal cellular activity, and it was shown to be beneficial for normal brain synaptic activity in the absence of neurotoxicity [25]. Moreover, the picomolar range concentrations of synthetic Aβ1-42 positively affect the synaptic transmission in vivo. Yet, nanomolar range concentrations of Aβ1-42 appear to be neurotoxic, involving detrimental effects as impairment of synaptic transmission [26,27]. The mechanism underlying Aβ–mediated synaptic dysfunctions is still unclear, but it may be related to the reduction of postsynaptic density protein 95 (PSD95) levels and negative regulation of α-amino-3-hydroxy-5-methyl-4-isoxazolepropionic acid (AMPA) and N-methyl-D-aspartate (NMDA) glutamatergic receptors [28]. Aβ also contributes to BDNF signaling disruption, impairing neuronal plasticity, thus affecting learning and memory processes [29]. Furthermore, Aβ mediates the generation of reactive oxygen species (ROS) that rapidly react with lipids, proteins, or deoxyribonucleic acids (DNA) determining lipid peroxidation, protein, and DNA oxidation [30]. Besides, the Aβ peptide induces inflammatory responses through microglial cell activation leading to the release of pro-inflammatory cytokines, which may increase neuronal degeneration and neuronal synapses loss [31,32].

In this study, an amyloidosis rat model was used in an attempt to demonstrate the memory-enhancing effects of OmEO, which, because of the Aβ1-42-related perturbation, may be clearer to determine, as opposed to studying non-challenged animals.

## 2. Materials and Methods

### 2.1. Plant Material

The aerial flowering parts of *Origanum majorana* L. (Lamiaceae) were collected during August 2019 from green crops within the Agricultural Research and Development Unit of Secuieni, Neamt County, Romania. A voucher specimen was botanically authenticated and preserved for ready reference at the Department of Pharmacognosy, Faculty of Pharmacy, University of Medicine and Pharmacy “Gr. T. Popa”, Iasi, Romania. The essential oil was obtained through 3 h hydro-distillation of fresh plant material using a Clevenger type equipment. Anhydrous sodium sulfate was used to dry the essential oil, removing the water from the distillate. It was then stored in amber vials at a temperature of 4 °C for further examination. The essential oil extraction yield was 0.34% (*v*/*v*).

### 2.2. Gas Chromatography-Mass Spectrometry (GC-MS) Analysis

The analysis of the essential oil was done using a gas chromatograph (Agilent 6890N, Agilent Technologies, Santa Clara, CA, USA) with 5975 inert XL mass selective detector (Agilent Technologies, Santa Clara, CA, USA) and flame ionization detector (GC-FID) equipped with a 30 m × 0.25 mm × 0.25 μm column (5%-phenyl)-methylpolysiloxane (HP 5MS, Agilent Technologies, Santa Clara, CA, USA); the injector temperature was 250–280 °C; increasing 4 grades/min; as mobile phase was used helium 1mL/min; split ratio 1:100; sample volume 0.2 µL. The retention indices (RI) were calculated against hydrocarbon (C4–C40) standards. Identification of the essential compounds was carried out by comparing retention times (RT), retention indices (RI), and mass spectra with those obtained from Wiley libraries [33].

### 2.3. Animals

Twenty-eight adult male Wistar rats were purchased from Cantacuzino Institute (Bucharest, Romania) with an average weight of 250 g (±80 g) and an age of 3 months. Animals were housed in 1500 U polysulfone cages (480 × 325 × 210 mm) (Tecniplast, Buguggiate, Italy) under standard laboratory conditions (22 °C room temperature, 12 h light/12 h dark cycle) with ad libitum access to water and food. All animal experiments were carried out according to the European Communities Council Directive (Directive 2010/63/EU) as well as the “Principles of Laboratory Animal Care” (NIH publication No. 85-23) concerning the protection of animals used for scientific and experimental purposes with approval of the Ethical Committee (No. 15309/22.07.2019).

### 2.4. Experimental Protocol for Generating the AD Rat Model

**Figure 1:**
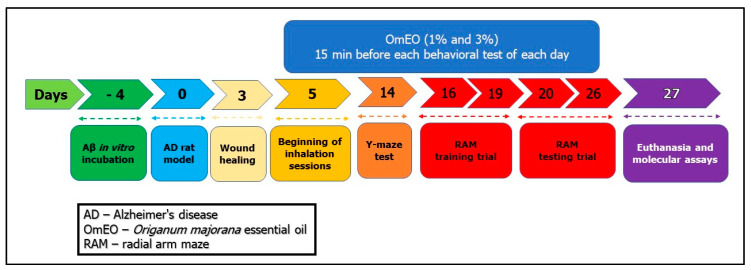
The experimental design of drug treatment and behavioral measurements.

Rats were anesthetized by intraperitoneal (i.p.) injection of the sodium pentobarbital solution (50 mg/kg, b.w., i.p., Sigma-Aldrich, Darmstadt, Germany) and mounted in a stereotaxic device. An iodine solution (10%, Hach, Loveland, CO, USA) was used to wash the scalp of the animals before midline incision, and then a thin hole (0.75 mm diameter) was drilled (Parkside Microdrill, Germany) in the skull 1.5 mm unilaterally right from the bregma craniometric point. To induce the AD rat model as previously described [34], Aβ1-42-containing solution (1 mM, Sigma-Aldrich, Darmstadt, Germany, dissolved in 0.9% sterile saline solution) was incubated at 37 °C for 4 days (Figure 1: day 4). Afterward, a volume of 4 μL of Aβ1-42 solution was intracerebroventricularly (i.c.v.) delivered for 4 min (flow rate 1 μL/min) by a Hamilton microsyringe 7.4 mm ventral from the surface of the cortex, according to the stereotaxic atlas (Figure 1: day 0) [35]. The syringe needle was left in place for another 5 min before being slowly removed. The control animals (sham-operated rats) received 4 μL of 0.9% sterile saline solution instead of the Aβ1-42 solution. Finally, the incisions were sutured, and the animals received a wide-spectrum antibiotic (as powder that was spread along the suture) before being returned to their home cages to recover. Postoperatively, the animals were individually housed in cages for 3 days for incision healing (Figure 1), having free access to water and food.

### 2.5. Drug Treatment and Experimental Design

The rats were divided into 4 groups (*n* = 7 animals/group): the control group (sham-operated) (I), the Aβ1-42 (1 mM, Sigma-Aldrich, Darmstadt, Germany), alone-treated group (II), and the Aβ1-42 receiving OmEO by inhalation groups (Aβ1-42+ 1%OmEO (III) and Aβ1-42+ 3%OmEO (IV)). InVivoStat and R-based statistical packages were used to confirm that *n* = 7 animals per group is appropriate [36]. Moreover, considering a significance level of 0.05, the power to detect a 20% biologically relevant change from control is 93%. Sham and Aβ1-42 groups received 1% Tween 80 solution through inhalation. OmEO was diluted with 1% Tween 80 (*v*/*v*). OmEO exposure (200 μL, either 1% or 3%) was via an electronic vaporizer (KBAYBO, China). Rats were treated by inhalation with OmEO (1% and 3%), which started at day 5 post-surgery (Figure 1) and lasted till the end of the behavioral tests (21 days), for 15 min each day. As for the concentrations indicated in the pharmacological tests, 1% essential oil, commonly used in aromatherapy, and a concentration of 3% were used to increase the effects, as we previously stated [34]. On day 27 (Figure 1), all the rats were euthanized with overdoses of sodium pentobarbital (150 mg/kg, b.w., i.p., Sigma-Aldrich, Darmstadt, Germany) and brain regions were excised on ice. From three randomly selected rats per group, the excised hippocampi were collected in RNA Save solution (Biological Industries, Beit-Haemek, Israel) and stored at −80 °C for further RNA isolation and qRT-PCR analysis. From the other three randomly selected rats per group, the excised hippocampi were immediately stored at −20 °C for further biochemical parameter assessments. From the remaining one rat per group, the hippocampi and the surrounding cerebral cortices were excised and immediately stored at −80 °C for proteome analysis (for surgery details, see Supplemental File 1).

### 2.6. Biochemical Parameters Assay

For the assay of the biochemical parameters, the frozen hippocampi were allowed to thaw for 2 min at room temperature upon which they were individually homogenized (1:10) in ice-cold 0.1 M potassium phosphate buffer (pH 7.4), 1.15% KCl by using a Potter Homogenizer (Cole-Parmer, Vernon Hills, IL, USA). The homogenates were centrifuged at 960× *g* for 15 min at 4 °C. The supernatant was used for the estimation of protein carbonyl and malondialdehyde (MDA) levels following the methods described [34]. The total protein content was determined using a bicinchoninic acid (BCA) protein assay kit (Sigma-Aldrich, Darmstadt, Germany) according to a method previously described [37].

### 2.7. Apoptotic State Evaluation

A cell death detection ELISA kit (Roche Diagnostics, Mannheim, Germany) was used for quantification of histone-associated DNA fragments. Following the protocol provided by the manufacturer, the reconstituted biotinylated anti-histone antibody was incubated for 1 h and then washed from the microplate (MP) module. For the non-specific binding sites blocking, the incubation solution was added on the MP module and kept for 30 min. Following a washing step, the sample solution, containing 1:20 dilution of the centrifuged hippocampal homogenate prepared for biochemical assays, was incubated on MP module for 1 h and 30 min. Then, the sample solution was washed, and the reconstituted anti-DNA- peroxidase (POD) antibody was incubated for 1 h and 30 min. Following the washing of unbound anti-DNA-POD antibody, the substrate solution was added on the MP module and incubated on a shaker plate for 10 min. The POD retained in the imunocomplex was photometrically determined using a microplate reader (BioTek, Winooski, VT, USA). The enrichment factor, reflecting n-fold apoptosis, was calculated as the absorbance of the sample/absorbance of negative control, where the negative control was represented by the sham-operated rats.

### 2.8. RNA Isolation and Hippocampal Real-Time Quantitative PCR (qRT-PCR)

The hippocampi stored in RNA Save solution in frozen state were allowed to thaw for 2 min at room temperature and 45 mg from each hippocampus was collected. For the collected hippocampal tissues, SV Total RNA Isolation System kit (Promega, Madison, WI, USA) was applied in order to isolate and purify the total ribonucleic acid (RNA), according to the manufacturer’s instructions and the protocol previously described [38]. For quantifying the expression of genes of interest, reverse transcription and amplification by real-time quantitative PCR (RT-qPCR) were performed using a GoTaq^®^ 1-Step RT-qPCR System (Promega, Madison, WI, USA) on a 5-plex HRM Rotor-Gene 6000 (Corbett, CA, USA) real-time PCR. The reaction mixture contained 10 µL GoTaq^®^ Probe qPCR Master Mix (1X) (Promega, Madison, WI, USA), 0.4 µL GoScript™ RT Mix for 1-Step RT-qPCR (50×), 2µL Forward primer, 2µL Reverse primer, 4 µL RNA solution, and 1.6 µL Nuclease-Free Water.

For the absolute quantification of two transcripts, BDNF and IL1β, primers with the following sequences were used: BDNF forward and reverse primer (F: 5′-ATT ACC TGG ATG CCG CAA AC-3′; R: 5′-TGA CCC ACT CGC TAA TAC TGT-3′, 101 bp product size) (M98820.1); IL1β forward and reverse primer (F: 5′- AGC ACC TTC TTT TCC TTC ATC TT-3′, R: 5′-CAG ACA GCA GGC ATT TT-3′, 144 bp product size) (M61178.1). The reaction thermic profile consisted of reverse transcription step—15 min (37 °C); RT inactivation/Hot-Start activation—10 min (95 °C); 40 cycles of 3-step qPCR: 10 s (95 °C)—denaturation, 30 s (60 °C)—alignment and data collection (Green channel—BRYT Green^®^ dye, Madison, WI, USA), 30 s (72 °C)—elongation; and dissociation step (from 60–95 °C). Rotor-Gene Q-Pure Detection Software v. 2.2.3. (Qiagen, CA, USA) was used for absolute quantification of BDNF and IL1β expression levels.

### 2.9. Proteomics Pilot Experiment

#### 2.9.1. Generation of Peptide Solutions for Proteome Analysis

Three pieces of frozen brain tissue containing the hippocampi (between 238 mg and 321 mg) were used to produce crude protein extracts. Each piece was from one specimen, to represent the experimental groups Aβ (1–42) (II), Aβ (1–42) + 1%OmEO (III), and Aβ (1–42) + 3%OmEO (IV). Each frozen brain tissue piece was thawed for 2 min at room temperature, weighed, and wetted with lysis buffer consisting of 1% sodium deoxycholate (SDC), 20 mM dithiothreitol (DTT) in 50 mM ammonium bicarbonate (ABC) and protease inhibitor cocktail Complete^TM^ (Roche, Mannheim, Germany) [39]. The added volume of lysis buffer (in µL) with respect to brain tissue weight (in mg) was in the 9:1 ratio, as described [40]. Brain tissue pieces and lysis buffer mixtures were transferred into a dounce homogenizer (Kontes Glass Co, Vineland, NJ, USA), and tissues were disrupted within 10 passes. To complete homogenization, suspensions were transferred into new 15 mL Falcon tubes, heated at 95 °C for 5 min in a water bath, followed by double ultra-sonication (Sonorex Super RK 31H, BANDELIN, Berlin, Germany), each for 15 s, interrupted for 2 min. Then, 50 mM ABC was added to each suspension in the same volume as the respective lysis buffer volume to reach a final concentration of 0.5% SDC. Next, the extracts were shaken for 10 min at room temperature and centrifuged at 16,000× *g* for 10 min at 20 °C. After centrifugation, the supernatants (crude protein extracts; volumes were between 4 mL and 6 mL) were aliquoted into 0.5 mL portions and transferred into new 1.5 mL Eppendorf tubes. For protein concentration determination, Bradford assay was used as described [41], due to its compatibility with reducing agents, such as DTT. Taking aliquots of crude protein extracts (300 µL to 400 µL), the reduced proteins (1 mg, each) were alkylated at room temperature in the dark for 20 min by adding 9 µL to 12 µL of 0.5 M iodoacetamide solution, dissolved in 50 mM ABC. The digestion of reduced and alkylated proteins was then performed overnight at 37 °C by adding 100 µL of trypsin solution (Promega, Madison, WI, USA; dissolved in 50 mM ABC) with an enzyme to substrate ratio of 1:100 (*w*/*w*). After digestion, peptide mixtures were centrifuged, supernatants (300 µL to 400 µL) were collected in separate 2 mL Eppendorf tubes, and phase transfer-assisted removal of SDC [42] was performed as previously described [43]. Ethyl acetate (in the same volume as the volume of the respective peptide mixture) and 25% trifluoroacetic acid (1/50 of the respective ethyl acetate volume) were added. The peptide mixtures were rigorously shaken for 2 min and subsequently centrifuged at 12,000× *g* for 10 min to obtain aqueous and organic phases. The aqueous phase of each peptide mixture was collected using a gel loading tip and reduced in volume to final volume of around 100 µL, each using a centrifugal evaporator (Speedvac RVC 2-25 CD plus, Martin Christ GmbH, Osterode am Harz, Germany). The peptide concentrations were measured using the Qubit assay as described [44], followed by desalting with OASIS cartridges [45]. Peptide concentrations of desalted peptide solutions were measured using the Qubit assay [44].

#### 2.9.2. Mass Spectrometric Proteome Analysis

Mass spectrometric analysis of peptide mixtures was performed in duplicate on a Synapt G2S mass spectrometer (Waters, Manchester, UK) using Masslynx version 4.1, coupled to a nanoAcquity UPLC system (Waters, Manchester, UK) via a NanoLockSpray ion source using a PicoTip Emitter (New Objective, Woburn, MA, USA) as described [41,46]. Mobile phase A contained 0.1% formic acid in water, and mobile phase B contained 0.1% formic acid in acetonitrile. Peptide solutions containing approx. 70 ng of peptides were supplemented with 40 fmol of Hi3 ClpB_ECOLI standard (Waters, Manchester, UK) for absolute quantitation. Peptides were trapped and desalted using a precolumn (nanoACQUITY UPLC Symmetry C18, 5 µm, 180 µm × 20 mm) (Waters, Manchester, UK) at a flow rate of 10 µL/min for 4 min with 99.9% A. Peptides were separated on an analytical column (ACQUITY UPLC HSS T3, 1.8 µm, 75 µm × 200 mm) (Waters, Manchester, UK) at a flow rate of 300 nL/min using a gradient from 3% to 35% B over 120 min. As a reference compound, 100 fmol/μL [Glu1]-fibrinopeptide B was delivered at 500 nL/min to the reference sprayer of the NanoLockSpray source. The SYNAPT G2S instrument was operated in a data-independent mode [47], characterized by parallel fragmentation of multiple precursor ions in combination with ion-mobility separation as an additional dimension of separation (referred to as HDMS^E^) [48]. By executing alternate scans at low and elevated collision energy (CE) of each 0.5 s, information on precursor and fragment ions, respectively, was acquired. In low-energy MS mode, acquisitions were performed at constant CE of 4 eV, whereas drift time-dependent CE settings were applied in elevated-energy MS mode. The values of drift time-dependent CE settings were used as described [49]. Lock spray was acquired once every 30 s for a 1 s period. Mass spectrometric data were stored as raw data files.

#### 2.9.3. Database Assembly and Proteome Data Analysis

For the database search, a database containing 29,944 protein sequences of the *Rattus norvegicus* proteome (http://www.uniprot.org/; UniProt release 2020_06) appended with the sequences of ClpB_ECOLI (P63284) and porcine trypsin was used. Mass spectrometric raw data file processing, protein identification, and label-free quantification of identified proteins were performed using the Progenesis QI for Proteomics software package, version 4.1 (Nonlinear Dynamics, Newcastle upon Tyne, UK) as previously described [46]. The following search parameters were set: trypsin as digestion reagent, two missing cleavage sites allowed, carbamidomethylation of cysteines as a fixed modification, and oxidation of methionine residues as variable modification. The false discovery rate was set to 1%. At least three fragment ions were required for peptide identification, and at least six fragment ions corresponding to minimally two peptides were required for protein identification. Moreover, for peptide identifications, only peptides with two, three, and four positive charges were accepted if they were identified in at least two of the six measurement series. In addition, the peptides with a peptide score below 5.7, absolute mass error above 13 ppm, and less than 6 amino acid residues in length were excluded.

The mass spectrometry proteomic data have been deposited to the ProteomeXchange Consortium via the PRIDE [50] partner repository with the dataset identifier PXD021082.

### 2.10. Behavioral Analysis

#### 2.10.1. Y-maze

Spontaneous alternation behavior and hippocampus integrity were assessed through a single session Y-maze as previously described [51,52]. The Y-maze used in the present study was constructed of Plexiglas with the following dimensions: 25 cm high, 35 cm long, 10 cm wide of each arm, and an equilateral triangular central area. Fourteen days post-surgery (Figure 1) and 15 min from OmEO (1% and 3%) inhalation session, each animal was placed at the end of an arm and allowed to explore the maze for 8 min. Successive entry in each arm was interpreted as behavioral alternation. A blinded observer registered visited arms sequence corresponding to each animal. The spontaneous alternation percentage was calculated as (number of alternation/total entries − 2) × 100. Between trials, the Y-maze was washed with a 10% ethanol solution.

#### 2.10.2. Radial Arm Maze

Spatial memory performance was assessed through radial arm maze (RAM) as previously described [53]. Before the start of the test, the animals were subjected to a weight loss regime, until the body weight of the animals represented 85% of the initial weight. The food restriction represents an essential factor for an efficient arm-choice within RAM [54] and it has been shown that food-deprived rats performed RAM more efficiently than food-undeprived rats [55]. Despite restrictive access to food, the animals had free access to water. The maze comprising eight arms marked from 1 to 8 (48 cm × 12 cm), with a radial extension of 32 cm in diameter from the central area, had 50 mg food pellet at the end of arms 1, 2, 4, 5, and 7. The RAM was started on day 16 (Figure 1) and performed in two stages: a training stage (4 consecutive days) aimed at habituation and a testing stage (7 successive days). Each training session lasted 5 min, with groups of 3 or 4 animals being tested. Although in the training days, the food baits were spread all over the maze, in test days, only 5 arms contained baits placed at the end of each arm. To perform the test, each animal was centrally positioned in the maze allowing it to explore the maze and consume the baits from the 5 arms. Behavioral assessment was completed when the animal consumed all 5 baits or after 5 min. An animal was considered to have entered an arm when all four of its members were in that arm. For working memory and reference memory tasks, each rat was individually placed in the center of the maze 15 min after inhaling OmEO (1% and 3%). Determinations were carried out by (i) assessing the number of working memory errors (getting inside an arm that contains food pellet, but earlier stepped into) and (ii) calculating the number of reference memory errors (calculating animal enters in an arm without food pellet). Spatial working memory represents the ability to hold process-specific information for a while, so that spatial responses can be performed flexibly from process to process. This type of memory is the basis of feeding behavior; thus, the subject remembering the arm just visited can adopt an effective search strategy. Spatial reference memory is represented by the ability to learn a consistent, fixed response to a spatial stimulus, being reflected by a constant association between the respective spatial location and an outcome. Thus, the animal will have to learn the position of the three arms not reinforced with baits, these remaining constant [56]. Between trials, the radial arm maze was washed with a 10% ethanol solution.

### 2.11. Statistical Analysis

All data are expressed as the mean ± standard error of the mean (S.E.M.). GraphPad Prism 8.0 (GraphPad Software, Inc., San Diego, CA, USA) was used to perform statistical analyses through a one-way analysis of variance (ANOVA) followed by Tukey’s *post hoc* multiple comparison test, considering treatment as a factor. A *p*-value of < 0.05 was considered significant. For protein analysis, Origin Pro 2016 (Originlab Co, MA, USA) and Excel methods of calculation were used.

## 3. Results and Discussions

### 3.1. Phytochemical Profile of the Origanum Majorana Essential Oil

Using GC-FID and GC-MS, 37 chemical compounds were identified in the freshly prepared OmEO (altogether, about 94.34% of the volatiles) (Supplemental File 2). The chemical constituents identified included monoterpenes (90.42%), whereas sesquiterpenes (7.92%) were minor components. The analysis of OmEO showed terpinen-4-ol (23.52%), sabinene (12.59%), terpinolene (8.72%), linalool (5.94%), β-thujene (4.60%), phellandrene (4.35%), and α-terpineol (4.30%) as the most abundant volatile components. Based on these findings, our essential oil exhibits a chemical composition equal to those documented by other authors [57,58,59,60].

### 3.2. Proteome Analysis of the Rat Brains Containing Hippocampi

**Figure 2:**
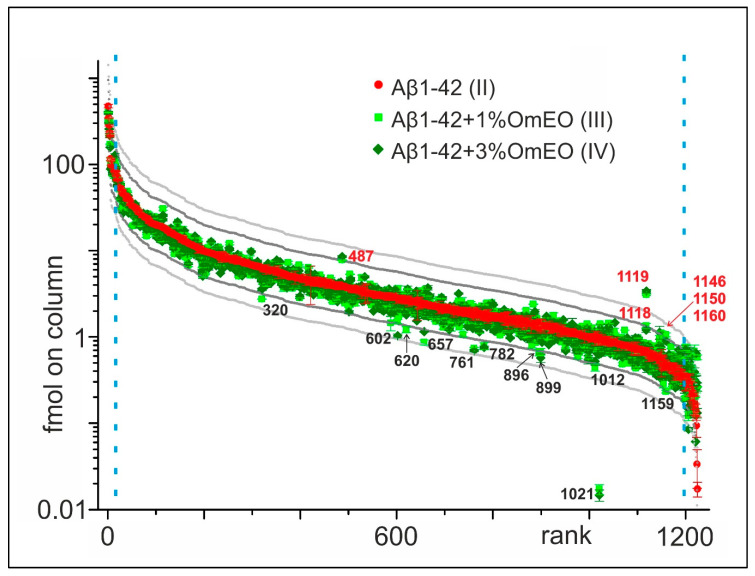
Abundance comparison of 1224 identified proteins in rat brain tissues. Ordering by abundance in Aβ1-42 (II; red dots). Protein abundances from Aβ1-42 and 1%OmEO (III; light green squares) and Aβ1-42 and 3%OmEO (IV; dark green diamonds) are overlaid. With numbers (red: above and black: below) are marked the proteins whose abundances were modulated by OmEO. The 2× threshold is represented by dark grey line and the 3× threshold by the light grey line. The blue vertical dashed lines mark borders in between which differential abundances were analyzed.

For this project, a data-driven research approach was followed using proteomics to generate an overview regarding the potential molecular effects of OmEO therapy, instead of a solely theory- or hypothesis-driven research approach [61]. Initially, 1224 proteins were identified in the rat brain tissue containing hippocampi (Supplemental File 3), which were ranked according to their relative abundances in the Aβ1-42 group (II) (Figure 2). Next, abundance profiles of the Aβ1-42 and 1%OmEO (III) and Aβ1-42 and 3%OmEO (IV) were superimposed to visualize abundance differences. Abundance differences were categorized by the 2× threshold line and the 3× threshold line, respectively. The most abundant 25 proteins and the least abundant 25 proteins were excluded from abundance comparisons.

It is tempting to regard the proteins above and below the 2× and 3× threshold lines as being of potential interest (Figure 2). However, considering the low number of animals per group, the abundance differences of those particular proteins should not be overemphasized, as statistical significance cannot be reached. Instead, the listed proteins in sum seemed to point to respective pathways/biological processes of interest, which then were tested for their potential roles with regard to OmEO-related effects by applying well-established functional assays (Table 1).

From the 17 proteins whose expression seemed influenced by OmEO administration (Table 1), 10 were regarded as representatives of biological processes, such as cognitive function, apoptosis, neuroinflammation, and oxidative stress, respectively. These biological functions were further targeted using well established biochemical/molecular assays one after the other (see below). The protein with the accession number Q5XIU9 is involved in adipose tissue development and, although *Origanum majorana* extract proved to be efficient in modulating lipid accumulation in liver and kidneys [63], we did not further assess this protein’s function since the biological process in which it is involved in was not the focus of this study. The remaining six proteins (Q08163, M0RBL8, P31232, P84083, Q5M821, and P01835) were assigned by Funrich to biological processes, such as energy consumption or cell motility. Although they indicated cellular activity in general, e.g., associated with healing processes and cellular regeneration, these functions were not further investigated.

### 3.3. Differential Activities of Biological Processes Determined by Functional Assays

#### 3.3.1. Neuroinflammation

Since the nuclear factor kappa-B (NF-κB) regulates transcription of a cohort of genes implicated in immune and inflammatory responses [64] and its activation involves three major signaling pathways: (i) the canonical pathway, triggered by TNFα or IL-1; (ii) the non-canonical pathway, elicited by CD40 ligand or lymphotoxin β; and (iii) atypical signaling pathways, initiated by DNA-damage [65], its role in neuroinflammatory processes [66] was of interest. IL-1β is a crucial mediator of inflammatory responses [67], and its quantification by RT-qPCR was regarded as the method of choice [68].

**Figure 3:**
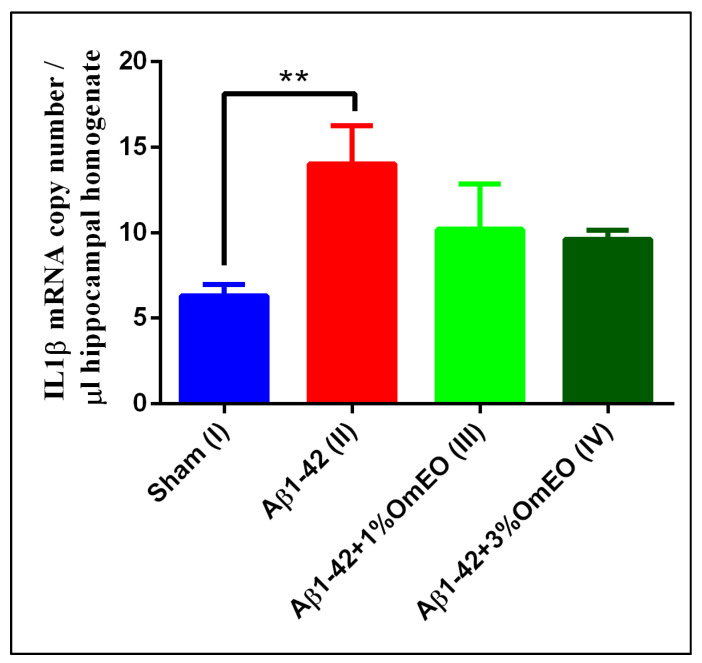
Effects of the inhaled *Origanum majorana* essential oil (1%OmEO (III) and 3%OmEO (IV)) on the IL-1β mRNA copy number estimated in the rat hippocampal homogenates of the Aβ1-42-treated rats. Values are means ± S.E.M. (*n* = 3 animals per group). Statistical significance was determined by one-way ANOVA. For Tukey’s *post hoc* analyses: Sham (I) vs. Aβ1-42 (II): ** *p* = 0.0032.

IL-1β mRNA copy numbers, which are related to inflammatory processes, were found significantly overexpressed in the Aβ1-42 pretreated group (II) vs. the sham-operated group (I) (*p* = 0.0032) (Figure 3). Yet, the inhaled OmEO attenuated only slightly the inflammatory response observed in Aβ1-42-pretreated rats, the minor changes induced by both concentrations of OmEO being statistically insignificant (*p* = 0.1107 for 1%OmEO (III) and *p* = 0.0635 for 3%OmEO (IV), respectively).

The IL-1β overexpression in response to experimentally induced neurotoxic stimuli is well documented [69]; thus, the increased expression of IL-1β induced by Aβ1-42 stands in general agreement with previous results. Furthermore, anti-inflammatory properties of OmEO were already mentioned in literature and sustained by in vitro studies [21]. Yet, our in vivo study found the potential of OmEO to reduce inflammation to be questionable as statistical significance was not reached.

#### 3.3.2. Apoptosis

NF-κB is generally regarded as being anti-apoptotic. Yet, in certain contexts, NF-κB may as well act as a promotor of apoptosis, especially in response to cellular stress [70,71]. To test whether apoptosis was a biological process with significance to OmEO treatment-related effects, apoptosis was investigated using a specific cell death detection ELISA assay, as was previously demonstrated [72,73,74].

**Figure 4:**
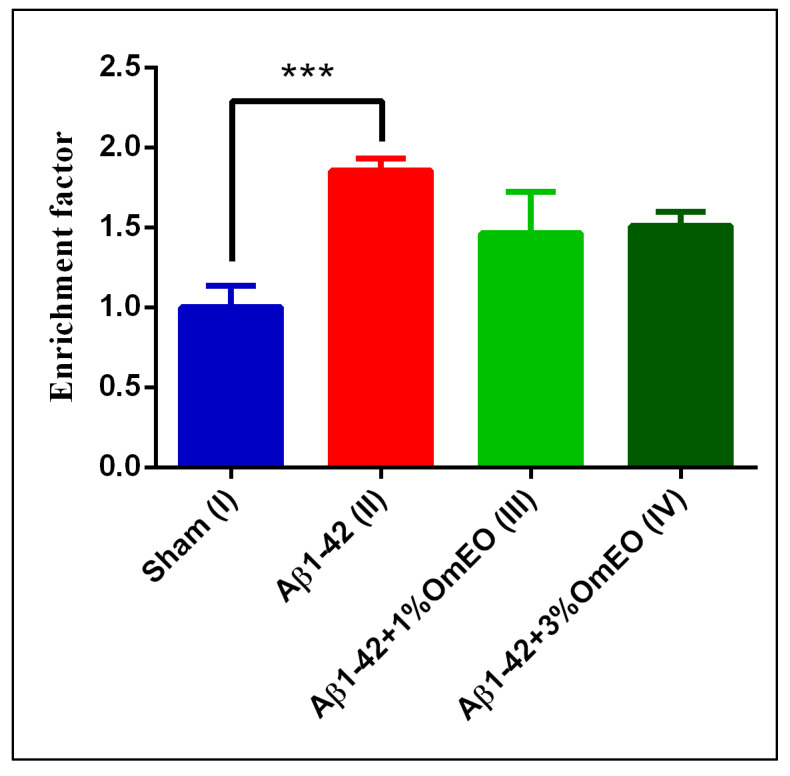
Effects of the inhaled *Origanum majorana* essential oil (1%OmEO (III) and 3%OmEO (IV)) on apoptotic state in the rat hippocampal homogenates of the Aβ1-42-treated rats. Values are means ± S.E.M. (*n* = 3 animals per group). Statistical significance was determined by one-way ANOVA. For Tukey’s post hoc analyses: Sham (I) vs. Aβ1-42 (II): *** *p* = 0.0008. The rate of apoptosis reflects n-fold nucleosomes enrichment and it is shown on the Y axis.

DNA fragmentation, associated to apoptotic processes, was found significantly elevated in the Aβ1-42 pretreated group (II) vs. the sham-operated group (I) (*p* = 0.0008) (Figure 4). Yet, OmEO inhalation determined only moderated reductions of DNA fragmentation (*p* = 0.0634 for 1%OmEO (III) and *p* = 0.1059 for 3%OmEO (IV), respectively).

Brain DNA damage naturally occurs during aging and exacerbates in different pathologies, such as AD. As shown in this study, Aβ1-42 administered to rats determined increased DNA fragmentation, indicating increased apoptosis. However, the specific intracellular signaling pathways by which Aβ triggers the apoptotic processes are poorly defined, even though several pathways were already proposed [75,76,77,78]. From our results, we deduce that OmEO ameliorated only to some extent the fragmentation of DNA. Therefore, the anti-apoptotic properties of OmEO cannot be asserted with statistical significance. Hence, we further investigated the efficiency of OmEO in counteracting other biological processes, such as oxidative stress as well as cognitive function (Table 1).

#### 3.3.3. Oxidative Stress

To test for oxidative stress responses, evaluation of lipid peroxidation and protein carbonylation was targeted. MDA, a secondary product of lipid peroxidation, is involved in multiple cellular processes and can promote intramolecular or intermolecular protein/DNA crosslinking, thus inducing profound alterations in the biochemical properties of a variety of biomolecules [79]. Protein carbonylation, an irreversible oxidative damage, most often leads to a loss of protein function, but may also lead to gain of function. In both cases, even small changes in carbonylation may determine significant changes in cellular function, affecting major signaling pathways [80,81].

**Figure 5:**
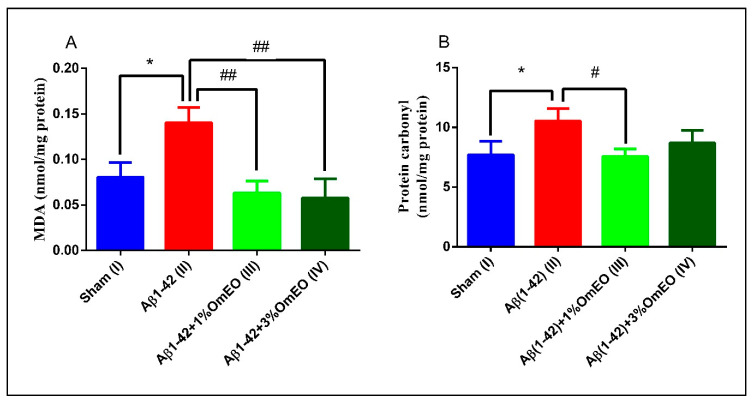
Effects of the inhaled *Origanum majorana* essential oil (1%OmEO (III) and 3%OmEO (IV)) on the MDA (**A**) and protein carbonyls (**B**) levels estimated in the rat hippocampal homogenates of the Aβ1-42-treated rats. Values are means ± S.E.M. (*n* = 3 animals per group). Statistical significance was determined by one-way ANOVA. For Tukey’s *post hoc* analyses: A. Sham (I) vs. Aβ1-42 (II): * *p* = 0.0110, Aβ1-42 (II) vs. Aβ1-42 + 1%OmEO (III): ## *p* = 0.0023 and Aβ1-42 (II) vs. Aβ1-42 + 3%OmEO (IV): # *p* = 0.0015; B. Sham (I) vs. Aβ1-42 (II): * *p* = 0.0283 and Aβ1-42 (II) vs. Aβ1-42 + 1%OmEO (III): # *p* = 0.0224.

Aβ1-42 pretreated rats (II) presented a significantly higher levels of MDA and protein carbonyls (*p* = 0.0110 and *p* = 0.0283) (Figure 5A,B) compared to the sham-operated rats (I), revealing the potential of Aβ1-42 single administration to induce oxidative stress in entire organ. Next, it was observed that the inhaled OmEO proved to be very efficient in counteracting the induced oxidative stress, significantly reducing MDA levels in Aβ1-42 pretreated rats (*p* = 0.0023 for 1%OmEO and *p* = 0.0015 for 3%OmEO, respectively) (Figure 5A). The efficiency of OmEO in oxidative stress reduction was substantiated by the results obtained from analyzing carbonyl contents of proteins (Figure 5B), where only 1%OmEO (III) significantly decreased the protein carbonylation process within the brains of Aβ1-42 pretreated rats (II) (*p* = 0.0224).

Our results are in accordance with those from literature, where different lines of evidence suggest that Aβ acts as a pro-oxidant [82], causing elevated levels of both MDA and protein carbonyls [83]. Previous studies revealed the reducing oxidative stress proprieties of OmEO in vitro [84] or in the context of nephrotoxicity, which was induced by different agents [85,86].

Of note, this in vivo study is the first which demonstrates the potential of OmEO to counteract oxidative stress in rat brain (hippocampus) which had been induced by administering a neurotoxic agent: Aβ1-42.

#### 3.3.4. Cognitive Function

RT-qPCR was used to analyze the expression of BDNF, and this widely distributed brain neurotrophin was selected to being assayed because of its involvement in critical regulatory roles of signaling pathways, which are involved in cognitive function-determining processes, such as developmental processes, neuroprotection, and synaptic plasticity [87,88] including short- and long-lasting synaptic interactions involved in the mechanism of memory and cognition.

**Figure 6:**
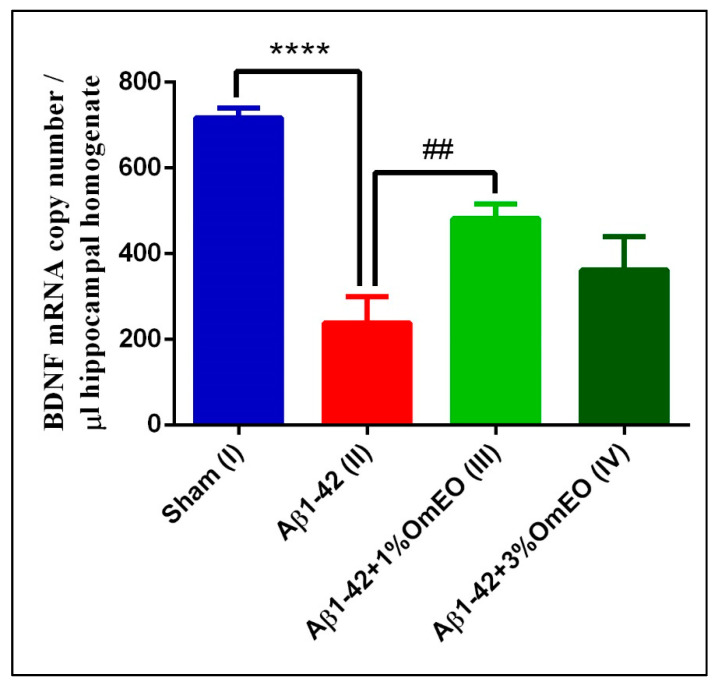
Effects of the inhaled *Origanum majorana* essential oil (1%OmEO (III) and 3%OmEO (IV)) on the BDNF mRNA copy number estimated in the rat hippocampal homogenates of the Aβ1-42-treated rats. Values are means ± S.E.M. (*n* = 3 animals per group). Statistical significance was determined by one-way ANOVA. For Tukey’s *post hoc* analyses: Sham (I) vs. Aβ1-42 (II): **** *p* < 0.0001 and Aβ1-42 (II) vs. Aβ1-42 + 1%OmEO (III): ## *p* = 0.0023.

When investigating brain-specific reactions onto OmEO-based aromatherapy, a significantly lower number of BDNF mRNA copies were determined in Aβ1-42 pretreated rats (II) compared to the sham-operated rats (I) (*p* < 0.0001) (Figure 6). In addition, it was observed that OmEO attenuated the damage induced by Aβ1-42 administration, as judged from a significant increase in BDNF expression being detected in the Aβ1-42 pretreated rats that inhaled the 1% concentration of the essential oil (III) (*p* = 0.0023). Yet, the 3% concentration of the OmEO (IV) was found to be somewhat less efficient in counteracting the harmful effects of Aβ1-42 (*p* = 0.08351) (Figure 6).

The impact of Aβ peptide on BDNF expression has been previously investigated by us [38] and others [89,90] and, as also shown in this study, BDNF expression was found impaired upon intracerebroventricular Aβ1-42 delivery. More importantly, this study provides direct evidence of the beneficial effect of OmEO inhalation regarding BDNF expression in an AD-rat model.

Particularly, the positive effects of OmEO on cognitive function-associated processes encouraged behavioral tests with animals that had received aroma therapy after administration of Aβ1-42.

### 3.4. Behavioral Tests

**Figure 7:**
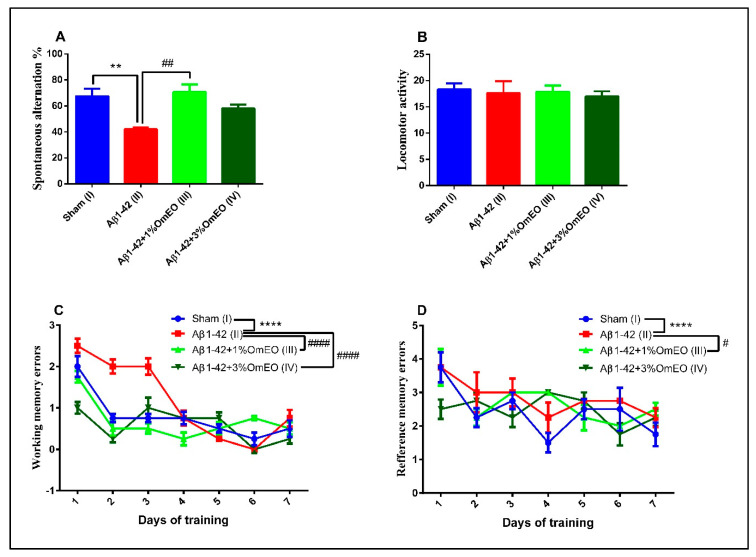
Effects of the inhaled *Origanum majorana* essential oil (1%OmEO (III) and 3%OmEO (IV)) on the spontaneous alternation percentage (**A**) and locomotor activity (**B**) in the Y-maze test and on the working memory errors (**C**) and reference memory errors (**D**) during seven-day training in the radial arm-maze test in the Aβ1-42-treated rats. Values are means ± S.E.M. (*n* = 7 animals per group). Statistical significance was determined by one-way ANOVA. For Tukey’s *post hoc* analyses: A. Sham (I) vs. Aβ1-42 (II): ** *p* = 0.0048 and Aβ1-42 (II) vs. Aβ1-42 + 1%OmEO (III): ## *p* = 0.0018; C. Sham (I) vs. Aβ1-42 (II): **** *p* < 0.0001; Aβ1-42 (II) vs. Aβ1-42 + 1%OmEO (III): #### *p* < 0.0001 and Aβ1-42 (II) vs. Aβ1-42 + 3%OmEO (IV): #### *p* < 0.0001; D. Sham (I) vs. Aβ1-42 (II): **** *p* < 0.0001 and Aβ1-42 (II) vs. Aβ1-42 + 1%OmEO (III): # *p* = 0.0142.

The Y-maze task was utilized to evaluate the short-term memory performance of rats that received different treatments, one-way ANOVA showing significant effects of these on spontaneous alternation behavior (F(3, 16) = 8.27, *p* = 0.0048). Single Aβ1-42 intracerebroventricular administration (II) caused a significant decrease in the spontaneous alternation behavior compared to the sham-operated control group (I) (*p* < 0.001) (Figure 7A). However, inhalation of the OmEO in both concentrations enhanced the performance of Aβ1-42 pretreated rats in the Y-maze test, significant improvements of the spontaneous alternation behavior being observed for the Aβ1-42 pretreated rats that inhaled 1%OmEO (III) (*p* = 0.0018), but not for those that inhaled 3%OmEO (IV) (*p* = 0.0950). The changes in the spontaneous alternation behavior cannot be attributed to the locomotor activity, as evidenced by the number of arm entries within the Y-maze task (Figure 7B).

In the radial arm maze task, two-way ANOVA analysis revealed major changes among the groups for both working memory performance (F(18, 112) = 43.98, *p* < 0.0001) (Figure 7C), as well as for reference memory performance (F(18, 112) = 9.29, *p* < 0.0001) (Figure 7D). As compared to the control group (I), the evaluation of the pretreated Aβ1-42 animals (II) was characterized by significant increments regarding the number of working memory errors (*p* < 0.0001) (Figure 7C), as well as the number of reference memory errors (*p* < 0.0001) (Figure 7D), proving that the single Aβ1-42 administration impaired the spatial memory. The inhaled OmEO in both concentrations (III and IV) determined a significant reduction of working memory errors in pretreated Aβ1-42 animals (*p* < 0.0001) (Figure 7C). On the other hand, reference memory acquisition was enhanced only in Aβ1-42 pretreated animals that inhaled 1%OmEO (III) (*p* = 0.0142) (Figure 7D). The inhalation of 3%OmEO (IV) did not improve the reference memory performance in Aβ1-42 pretreated animals (*p* = 0.9717) (Figure 7D).

These behavioral test results are in line with previous reports, which demonstrated the potential anti-AD effects of selected Lamiaceae plants [91]. Altogether, our study provides evidence that OmEO ameliorates Aβ-induced perturbations, such as memory deficits, and offers insights into the cellular and molecular mechanisms involved in the protective effects of this essential oil against Aβ-induced cognitive dysfunctions.

## 4. Conclusions

In this study, we first confirmed the chemical composition of OmEO, which was applied for analyzing the proteomes of AD model rats, in search of differences that might be displayed on the molecular level. Through this approach, four biological processes—neuroinflammation, apoptosis, oxidative stress, and cognitive functions modulated by neurotrophins—were brought to attention as possibly affecting rat brain (hippocampi) performance as a likely consequence of OmEO administration. Whether or not the suggested biological processes were of significance was validated by testing matching targets, which are accepted representatives of the biological processes of interest. In this study, we found that OmEO promotes cognitive functions and reduces brain oxidative stress in Aβ1-42-treated rats. Finally, we correlated the cellular and molecular results with behavioral data, proving that OmEO enhances memory function in Aβ1-42-treated rats.

## Figures and Tables

**Table 1:** List of proteins whose expression in brain was suspected to be modulated by OmEO, gene ontology grouping, and functional assay assignment.

Rank ^(a)^	No. ^(b)^	Protein Name ^(b)^	Cellular Component/Molecular Function/Biological Process ^(c)^	Assay ^(d)^
320	P30009	Myristoylated alanine-rich C-kinase substrate	postsynaptic cytoskeleton/calmodulin binding/positive regulation of neuron projection development	cognitive function
487	Q08163	Adenylyl cyclase-associated protein 1	cortical actin cytoskeleton/actin binding/actin cytoskeleton organization	n.a.
602	M0RBL8	Transcription elongation factor A (SII)-like 6	nucleus/WW domain binding/n.d.	n.a.
620	P31232	Transgelin	cytoplasm/actin filament binding/epithelial cell differentiation	n.a.
657	Q9EPH2	MARCKS-related protein	anchored component of presynaptic membrane/calmodulin binding/positive regulation of cell population proliferation	cognitive function
761	P84083	ADP-ribosylation factor 5	perinuclear region of cytoplasm/GTP binding/vesicle-mediated transport	n.a.
782	P60901	Proteasome subunit alpha type-6	cytoplasm/threonine-type endopeptidase activity/positive regulation of NF-κB transcription factor activity	apoptosis a/o neuroinflammation
896	Q6AY41	Cell cycle control protein 50A	Golgi apparatus and membrane/aminophospholipid flippase activity/positive regulation of neuron projection development	cognitive function
899	P34067	Proteasome subunit beta type-4	cytoplasm/threonine-type endopeptidase activity/proteasome-mediated ubiquitin-dependent protein catabolic process	oxidative stress
1012	Q6AXS5	Plasminogen activator inhibitor 1 RNA-binding protein	cytoplasm/mRNA 3′-UTR binding/regulation of apoptotic process	apoptosis
1021	P06302	Prothymosin alpha	nucleus/NF-κB binding/positive regulation of NF-κB transcription factor activity and negative regulation of apoptotic process	apoptosis a/o neuroinflammation
1118	Q5M821	Protein phosphatase 1H	n.d./protein serine/threonine phosphatase activity/positive regulation of pyruvate dehydrogenase activity	n.a.
1119	P01835	Ig kappa chain C region, B allele	n.d./n.d./n.d.	n.a.
1146	Q63941	Ras-related protein Rab-3	endomembrane system/GTP binding/peptidyl-cysteine methylation	oxidative stress
1150	P13084	Nucleophosmin	ribonucleoprotein complex/identical protein binding/positive regulation of NF-κB transcription factor activity	apoptosis a/o neuroinflammation
1159	Q5XIU9	Membrane-associated progesterone receptor component 2	membrane/steroid binding/adipose tissue development	n.a.
1160	P25093	Fumarylacetoacetase	n.d./metal ion binding/homogentisate catabolic process	oxidative stress

^(a)^ see Figure 2; ^(b)^ UniProt data base entry; ^(c)^ determined by Funrich [62]; n.d.: not determined; ^(d)^ investigated by standard biochemical and/or molecular biology assay; n.a.: not assayed.

## Supplementary Materials

The following are available online at https://www.mdpi.com/2076-3921/9/10/919/s1, Supplemental File 1. Excision of hippocampi and surrounding cerebral cortices for proteome analysis; Supplemental File 2 The phytochemical composition of OmEO; Supplemental File 3. Proteins were identified in the rat brain tissue containing hippocampi.
