# Supplementary material for: Memory-Enhancing Effects of Origanum majorana Essential Oil in an Alzheimer’s Amyloid beta1-42 Rat Model: A Molecular and Behavioral Study
Source: Antioxidants (Basel). 2020 Sep 26;9(10):919. doi: 10.3390/antiox9100919 (PMC7600529; doi:10.3390/antiox9100919)
Supplement: Supplementary file 1 [file antioxidants-09-00919-s001.zip › Supplementary File/PPA_2020_Rat Project_Supplemental_File_1.docx]

Excision of hippocampi and surrounding cerebral cortices for proteome analysis

The rat brain was extracted from the skull and rinsed with ice cold PBS (10 ml) to remove excess blood. Next, the brain was placed with the ventral side facing the pre chilled metal plate. The brain was cut bi-half into right and left hemisphere and the brain halves were gently separated. Each half was placed with the lateral side facing up. By cutting along the border of the cerebral cortex, the posterior part of the brain was removed. Then, the olfactory bulb and the frontal cortex were removed. The remaining tissue was flipped, facing up the medial side of each remaining hemisphere. The block of tissue covering the hippocampus was removed. Then the hippocampus and the surrounding cerebral cortex of each hemisphere were collected, shock-frozen by submerging in liquid nitrogen, and stored at -80^ο^C. A brain sample for proteome analysis contained both hippocampi and surrounding cerebral cortices corresponding to a rat brain.
