# Supplementary material for: Memory-Enhancing Effects of Origanum majorana Essential Oil in an Alzheimer’s Amyloid beta1-42 Rat Model: A Molecular and Behavioral Study
Source: Antioxidants (Basel). 2020 Sep 26;9(10):919. doi: 10.3390/antiox9100919 (PMC7600529; doi:10.3390/antiox9100919)
Supplement: Supplementary file 1 [file antioxidants-09-00919-s001.zip › Supplementary File/PPA_2020_Rat Project_Supplemental_File_2.docx]

Supplemental Table 1. The phytochemical composition of OmEO

| **No.** | **Compound** | **Abundance (%)** |
| --- | --- | --- |
| **MONOTERPENES** | | |
|  | α-pinene | 0.62 |
|  | **phellandrene** | **4.35** |
|  | β-pinene | 0.43 |
|  | myrcene | 1.15 |
|  | α- phellandrene | 0.49 |
|  | **terpinolene** | **8.72** |
|  | p-cymene | 2.85 |
|  | β-phellandrene | 2.95 |
|  | **β-thujene** | **4.60** |
|  | eucalyptol | 0.24 |
|  | β-ocimene | 0.17 |
|  | **sabinene** | **12.59** |
|  | terpineol | 1.72 |
|  | camphene | 0.08 |
|  | carene | 3.47 |
|  | **linalool** | **5.94** |
|  | isopropyl-1-methyl-2-cyclohexen-1-ol | 1.79 |
|  | cis-para-menth-2-en-1-ol | 1.20 |
|  | **terpinen-4-ol** | **23.52** |
|  | **α-terpineol** | **4.30** |
|  | estragole | 3.60 |
|  | isopiperitone | 2.60 |
|  | anethole | 0.36 |
|  | thymol | 0.09 |
|  | α-terpinene | 1.84 |
|  | geranyl acetate | 0.68 |
|  | limonene | 0.07 |
| **SESQUITERPENES** | | |
|  | caryophyllene | 3.81 |
|  | β-gurjunene | 0.18 |
|  | α-bergamotene | 0.32 |
|  | alloaromadendrene | 0.28 |
|  | copaene | 0.11 |
|  | α-selinene | 0.10 |
|  | γ-elemene | 2.04 |
|  | α--farnesene | 0.10 |
|  | (-)-spathulenol | 0.55 |
|  | caryophyllene oxide | 0.43 |
| **Total identified** | | **98.34** |
